# Current Use and Complementary Value of Combining *in Vivo* Imaging Modalities to Understand the Renoprotective Effects of Sodium-Glucose Cotransporter-2 Inhibitors at a Tissue Level

**DOI:** 10.3389/fphar.2022.837993

**Published:** 2022-02-21

**Authors:** Sjoukje van der Hoek, Jasper Stevens

**Affiliations:** Department of Clinical Pharmacy and Pharmacology, University of Groningen, University Medical Center Groningen, Groningen, Netherlands

**Keywords:** sodium glucose transporter 2, nephrology, diagnostic imaging, clinical pharmacology, chronic kidney disease, pathophisiology, renal circulation, hypoxia

## Abstract

Sodium-glucose cotransporter-2 inhibitors (SGLT2i) were initially developed to treat diabetes and have been shown to improve renal and cardiovascular outcomes in patients with- but also without diabetes. The mechanisms underlying these beneficial effects are incompletely understood, as is the response variability between- and within patients. Imaging modalities allow *in vivo* quantitative assessment of physiological, pathophysiological, and pharmacological processes at kidney tissue level and are therefore increasingly being used in nephrology. They provide unique insights into the renoprotective effects of SGLT2i and the variability in response and may thus contribute to improved treatment of the individual patient. In this mini-review, we highlight current work and opportunities of renal imaging modalities to assess renal oxygenation and hypoxia, fibrosis as well as interaction between SGLT2i and their transporters. Although every modality allows quantitative assessment of particular parameters of interest, we conclude that especially the complementary value of combining imaging modalities in a single clinical trial aids in an integrated understanding of the pharmacology of SGLT2i and their response variability.

## Introduction

The sodium-glucose cotransporter-2 (SGLT2), located in the proximal tubules of the kidneys, is responsible for 80–90% of glucose reabsorption. SGLT2 inhibitors (SGLT2i) increase renal glucose excretion and thus lower plasma glucose levels (Wright et al., 2007; DeFronzo et al., 2013). In contrast to other glucose-lowering agents, SGLT2i have been shown to improve renal and cardiovascular outcomes in patients with and without diabetes, including patients with chronic kidney disease (CKD) (Zinman et al., 2015; Neal et al., 2017; McMurray et al., 2019; Perkovic et al., 2019; Heerspink et al., 2020). The beneficial effects of SGLT2i cannot be solely attributed to improved glycemic control as the reduction in HbA1c is relatively modest; other pathways seem to be involved. Indeed, there is a growing interest in the renoprotective effects of SGLT2i on intraglomerular hypertension, improvement of hypoxia and stimulation of anti-inflammatory- and antifibrotic pathways (Sen and Heerspink, 2021).

Also, the response to SGLT2i, for example regarding HbA1c and albuminuria reduction, varies largely between individuals (Hwang et al., 2019; Heerspink et al., 2019a), and therefore, a considerable proportion of patients remain at risk of progressive renal function loss. To understand this response variability in patients, an integrative research approach is currently applied that focuses on the pharmacology of SGLT2i at tissue level.

In recent years, imaging modalities have seen great advances and are used for different organs and drugs, including development of new drugs that potentially protect organs such as the heart and kidney. As a result, imaging modalities are now increasingly used in nephrology. They allow *in vivo* assessment of physiological, pathophysiological, and pharmacological processes at kidney tissue level (Caroli et al., 2021; Klinkhammer et al., 2021). As such, imaging is becoming a powerful tool to improve our understanding of SGLT2i-pharmacology and is therefore anticipated to result in more effective treatment of the individual patient.

We highlight current work and opportunities of imaging modalities used in SGLT2 research to assess renal oxygenation and hypoxia, fibrosis and interaction between SGLT2i and their transporters.

## Renal Oxygenation and Hypoxia

Several pathological changes in CKD lead to an increased renal oxygen demand; for example, hyperglycemia, hyperfiltration and proximal tubular hypertrophy all lead to increased active tubular sodium reabsorption and therefore higher energy demand. On the other hand, renal perfusion (RP), defined as renal blood flow (RBF) per unit tissue, is compromised due to microvascular damage.

An imbalance between oxygen demand and RP results in hypoxia, widely believed to play an important role in the pathophysiology of kidney disease (Fine et al., 1998). Furthermore, microvascular dysfunction, or more likely, the broader process of hypoxia, is believed to be an important determinant of tubulointerstitial fibrosis, the hallmark of CKD and the best predictor of progressive renal disease (Eddy, 2005; Fine and Norman, 2008).

By blocking sodium- and glucose transport, SGLT2i are thought to reduce active sodium/potassium transport, resulting in reduced cortical oxygen demand (Kamezaki et al., 2018). In contrast, it is suggested that SGLT2i increase medullary energy expenditure due to a shift towards active sodium reabsorption processes in the thick ascending limb (O'Neill et al., 2015).

To understand changes in kidney oxygenation due to SGLT2 inhibition, it is of crucial importance to quantitate changes in cortical- and medullary kidney perfusion and oxygen consumption. Several magnetic resonance imaging (MRI) and positron emission tomography (PET) techniques are currently available and applied to measure different aspects of renal oxygenation.

### Renal Blood Flow and Perfusion

#### Dynamic Contrast-Enhanced MRI

Renal dynamic contrast-enhanced MRI (DCE-MRI) uses paramagnetic contrast fluids, usually a gadolinium chelate, to enhance the signal of water molecules. By kinetic modeling, the single kidney parameters RBF, GFR, and cortical and medullary blood volumes can be obtained. DCE-MRI is well-established to measure myocardial perfusion (Franks et al., 2021) and has also been validated in nephrology. Unfortunately, exposure to gadolinium contrast agents has been linked to nephrogenic systemic fibrosis in patients with advanced kidney disease (estimated glomerular filtration rate (eGFR) < 30 ml/min/1.73 m^2^) and therefore other methods are preferred in these patients (Bokacheva et al., 2008).

#### Phase-Contrast MRI

Phase-contrast MRI (PC-MRI) measures RBF in renal arteries by opposing gradient magnetic pulses that induce phase shifts in moving protons. When the kidney volume is also measured, e.g., by MRI, RP can be calculated and other parameters like renal vascular resistance can be derived (Liss et al., 2013; Khatir et al., 2019; Villa et al., 2020). Although primarily used in cardiology (Gatehouse et al., 2005; Nayak et al., 2015), PC-MRI recently showed reduced RBF in CKD patients vs. healthy controls (Khatir et al., 2014; Khatir et al., 2015). In Type I diabetes (T1DM) patients, PC-MRI showed an acute RBF decrease following a single dose of 50 mg dapagliflozin, although not significantly different from the control group (Laursen et al., 2021).

#### Arterial Spin Labeling MRI

Arterial spin labeling MRI (ASL-MRI) uses radiofrequency pulses to invert the magnetization of the water protons in blood that distribute into organs and thus quantifies tissue perfusion. Originating from brain research, its feasibility has now also been shown in nephrology. The contrast-to-noise-ratio is relatively low, but rapidly developing technical improvements may resolve this inconvenience. Cortical measurements have a good reproducibility over a wide range of renal functions, are correlated to eGFR and are validated in animals and man. However, at this moment, the reproducibility of medullary perfusion measurements is moderate to poor (Odudu et al., 2018). Cortical and medullary perfusion was reduced in Type II diabetes (T2DM) patients with normal eGFR vs. healthy controls, whereas perfusion decreased further with progression of nephropathy (Mora-Gutiérrez et al., 2017). Furthermore, cortical perfusion was consistently lower (28%–50%) in CKD- and diabetic kidney disease (DKD) populations (Gillis et al., 2016; Li et al., 2017). ASL-MRI found no acute change in perfusion after a single dose of dapagliflozin in T1DM patients (Laursen et al., 2021). Also, in newly diagnosed T2DM patients, perfusion did not change after a 24-weeks treatment with 100 mg canagliflozin, despite a persistent eGFR-reduction (Zhou et al., 2021). ASL-MRI is useful to investigate if SGLT2i improve hypoxia by changes in cortical perfusion in patients with reduced kidney function. Indeed, a study in T2DM patients assessing chronic effects of SGLT2i on multiple MRI parameters, including RP using ASL-MRI, is currently ongoing (NCT04027530).

#### Radio-Oxygenated Water

Radio-oxygenated water (H_2_^15^O) infusion followed by PET-imaging is the preferred method for assessment of regional cerebral (Ssali et al., 2018) and myocardial blood flow (Williams et al., 2017) and is now also being used to measure RBF (Alpert et al., 2002; Juillard et al., 2002; Päivärinta et al., 2018). Using H_2_^15^O, RBF was shown to be reduced in CKD patients vs. healthy controls (Alpert et al., 2002). Separate measurement of cortical-vs. medullary RBF is challenging due to the complex vascular anatomy in the medulla. Interestingly, H_2_^15^O administration followed by co-injection of glyceryl nitrate or N^ω^-monomethyl-l-arginine provides an index of the medullary blood flow (Damkjær et al., 2010). As this study was executed in 9 volunteers, method validation awaits. So far, studies with SGLT2i have not been performed with this method, although it is an attractive method to study changes in RBF.

### Oxygen Consumption

#### [^11^C]-Acetate PET

[^11^C]-Acetate PET imaging allows estimation of renal oxygen consumption. After infusion, [^11^C]-acetate is metabolized by renal cells and ^11^CO_2_ is formed. Kinetic modeling provides the [^11^C]-acetate turnover rate, which is indicative for the extent of oxidative metabolism. A validation study in pigs showed that [^11^C]-acetate turnover, as measured by its cortical clearance, was strongly correlated with alternative measures of renal oxygen consumption (Juillard et al., 2007). Good reproducibility of [^11^C]-acetate turnover was shown in patients with different renal diseases vs. healthy subjects and, importantly, a reduced [^11^C]-acetate turnover in CKD patients (Shreve et al., 1995). Furthermore, the initial renal uptake of [^11^C]-acetate is a measure for RBF, as shown by H_2_^15^O-based comparison in healthy individuals (Normand et al., 2019). Although widely used in cardiology (Stendahl and Sinusas, 2020; Oldgren et al., 2021), [^11^C]-acetate PET is scarcely used in nephrology, despite its great potential to investigate the effects of SGLT2i on renal oxygen consumption. One ongoing trial assesses the effects of SGLT2i with different imaging modalities including [^11^C]-acetate PET (NCT04027530).

### Renal Oxygenation

#### Blood Oxygenation Level-Dependent MRI

Blood-oxygenation-level-dependent MRI (BOLD-MRI) uses the difference in magnetic properties of oxygenated vs. deoxygenated hemoglobin (Pauling and Coryell, 1936). Method validation has been performed in pig and the reproducibility has been assessed in healthy volunteers and CKD patients (Li et al., 2004; Pedersen et al., 2005; Simon-Zoula et al., 2006; Khatir et al., 2014). The first BOLD-MRI studies evaluating renal oxygenation showed conflicting results. Nowadays, relationships between CKD and (cortical) renal hypoxia are consistent due to method standardization and corrections for confounding factors like hydration status, salt intake, co-medication and factors affecting the oxygen-hemoglobin dissociation curve like temperature and pH (Pruijm et al., 2018a; Hall et al., 2018). Importantly, increases in MRI field strength have resulted in better discrimination between cortical and medullary oxygenation (Prasad et al., 2015; Milani et al., 2017). BOLD-MRI has thus emerged as a standard method for measurement of kidney oxygenation that predicts eGFR decline in CKD and DKD populations (Pruijm et al., 2018b; Zhou et al., 2018; Sugiyama et al., 2020; Li et al., 2021). In apparent healthy populations, the predictive value of early hypoxia assessed by BOLD MRI on eGFR decline, which may aid in the design of interventional studies, is yet to be determined. Three studies have assessed SGLT2i effects on renal oxygenation by BOLD-MRI. No acute or chronic changes in cortical- or medullary oxygenation were found in healthy individuals receiving a daily dose of 10 mg empagliflozin (Zanchi et al., 2020). However, in a placebo controlled randomized crossover trial in T1DM patients with albuminuria receiving a single 50 mg dapagliflozin dose, an acute improvement in cortical oxygenation was reported (Laursen et al., 2021). In a T2DM population cortical- and medullary oxygenation increased by 22% and 29% respectively after 24-weeks treatment with 100 mg canagliflozin (Zhou et al., 2021). Interestingly, based on combining BOLD-MRI and ASL-MRI, the last two studies suggest that SGLT2i improve renal oxygenation without increasing RBF. One ongoing trial assesses SGLT2i effects on renal oxygenation using BOLD-MRI as main outcome (NCT04027530).

### Fibrosis

SGLT2i improve fibrosis-associated biomarkers in DKD patients (Heerspink et al., 2019b). Whether SGLT2i directly reduce fibrosis or that this is a result of anti-inflammatory effects, is not known (Terami et al., 2014). Also, *in vivo* data about antifibrotic properties of SGLT2i in human are lacking.

#### Diffusion-Weighted MRI

Diffusion-weighted magnetic resonance imaging (DWI-MRI) applies magnetic field gradients to renal tissue that displace water molecules. Kinetic modeling provides an apparent diffusion coefficient (ADC), which relates to water movement and DWI-MRI is therefore considered sensitive to changes in the renal interstitium, for example, due to renal fibrosis or edema, in RP and water handling in the tubular compartment (Caroli et al., 2018). Several studies report reduced renal water movement in CKD and T2DM patients vs. healthy controls and strong correlations between ADC and eGFR and CKD stage (Lu et al., 2011; Cakmak et al., 2014; Zhao et al., 2014). DWI-MRI comparison with renal biopsy confirmed correlation between lower cortical/medullary water movement and severity of fibrosis in CKD (Inoue et al., 2011; Zhao et al., 2014)**.** However, one study contradicts the correlation between fibrosis and eGFR, despite a similar relationship between fibrosis and ADC (Berchtold et al., 2020). Nonetheless, these studies show the potential of DWI-MRI to detect effects of SGLT2i on fibrosis earlier than eGFR. An observational cohort study in 500 T2DM patients is ongoing to assess whether DWI-MRI parameters can also be used as biomarkers in DKD (Gooding et al., 2020).

### SGLT2 Function and Inhibition

The glucose reabsorption capacity of the kidneys is increased during hyperglycemia. Some explanations have been proposed for this phenomenon, including hyperfiltration and tubular growth. Whether SGLT2 expression is increased is unknown (Vallon and Thomson, 2020). Several animal models of diabetes show an increased expression SGLT2 mRNA (Freitas et al., 2008; Tabatabai et al., 2009). However, the limited data regarding human glucose transporter expression is conflicting. One study reports increased SGLT2 expression as measured by quantitative real time PCR and immunohistochemistry in kidney biopsies from T2DM patients (Wang et al., 2017), whilst another did not (Solini et al., 2017). A third study reported an increased SGLT1-but not SGLT2 mRNA expression in T2DM kidney biopsies (Norton et al., 2017). Although kidney biopsy is the gold standard for kidney disease diagnostics, the sample taken may not be representative for the entire kidney, particularly in patchy diseases occurring in CKD. Renal imaging has the advantage of analysing the entire organ. *In vivo* data regarding SGLT2 expression are lacking. Also, little is known about kidney SGLT2i exposure and its interaction with SGLT2. To investigate changes in glucose transporter expression in kidney disease and understand the relationships between SGLT2i target exposure, receptor density and interaction with SGLT2, several radiolabeled substrates are currently used.

#### Positron Emission Tomography Imaging With Radiolabeled Substrates

2-Deoxy-2-[^18^F]-fluoro-D-glucose is widely used in oncology and inflammation, has a high affinity for GLUTs, but a low affinity for SGLTs (Sala-Rabanal et al., 2016) and is therefore not a very suitable PET-tracer to quantitate SGLT2 function *in vivo*. However, in T2DM patients, 2-weeks treatment with empagliflozin or dapagliflozin reduced 2-deoxy-2-[^18^F]-fluoro-D-glucose reabsorption by 10%, simultaneously confirming the low affinity for SGLT2 (Rasul et al., 2020). Several more specific tracers have been developed, although none seem highly SGLT2-specific. a-Methyl-4-[^18^F]-fluoro-4-deoxy-D-glucopyranoside is a high affinity SGLT1-substrate, a medium affinity SGLT2-substrate and a very low affinity GLUT-substrate, whereas 4-deoxy-4-[^18^F]-fluoro-D-glucose is substrate for both GLUTs and SGLTs (Sala-Rabanal et al., 2016). Mitsuoka et al. used the SGLT1 and SGLT2-specific substrate [^11^C]-methyl-d-glucoside to show an ipragliflozin-dose-related inhibition of [^11^C]-methyl-d-glucoside reabsorption (Mitsuoka et al., 2016). This shows that radiolabeled substrates can quantitate the glucose blocking potential of SGLT2i, but the individual contribution of SGLT1 and SGLT2 cannot be distinguished. To attain specific knowledge on SGLT2 expression, function or specific binding of SGLT2i, radiotracers with a high specific affinity for SGLT2 are needed.

#### Positron Emission Tomography Imaging With Radiolabeled Drugs

Compounds known to have a high specific affinity for SGLT2 are SGLT2i. As such, 4-[^18^F] fluoro-dapagliflozin was developed; a PET tracer of dapagliflozin where the 4-OH group is replaced by the radioactive isotope ^18^F. Animal studies showed specific affinity for SGLT2 and renal reabsorption (Ghezzi et al., 2017). However, due to the addition of an [^18^F]-fluoro group to dapagliflozin, 4-[^18^F] fluoro-dapagliflozin does not retain the original molecular structure of the marketed drug dapagliflozin. These results may therefore not fully reflect the pharmacokinetic and pharmacodynamic properties of dapagliflozin.

Recently, [^18^F]-canagliflozin, an isotopologue of canagliflozin, was developed (Van der Hoek et al., 2021). Since this tracer does retain the original structure of canagliflozin, it can be used to quantify canagliflozin distribution. Moreover, when used in canagliflozin-blocking experiments, [^18^F]-canagliflozin can be used to quantitate SGLT2 density and binding *in vivo*. An [^18^F]-canagliflozin feasibility study to non-invasively quantify the tissue canagliflozin distribution and SGLT2 density in T2DM patients is currently ongoing (Netherlands Trial Register; NL7707).

## Discussion

The mechanisms underlying the beneficial effects and response variability of drugs acting in the kidneys of CKD patients are incompletely understood. Imaging modalities are increasingly being used in nephrology to fill this knowledge gap. Also in the field of SGLT2i there is an increase in the use of these techniques and some exciting clinical trials are ongoing that should provide unique insights into the underlying pharmacology.

Impaired renal oxygenation is widely believed to affect the pathophysiology of CKD (Fine et al., 1998). Several imaging modalities are well capable of quantitating the effects SGLT2i have on different aspects of oxygenation. For this purpose, BOLD-MRI is a powerful tool as it measures cortical and medullary oxygenation separately and predicts eGFR decline. It may be used to enrich SGLT2i trials and, in combination with other imaging modalities or biomarkers, prove to be a predictive tool for detecting patients at risk for CKD progression (Pruijm et al., 2018a). However, oxygenation is the net balance between oxygen delivery and consumption that BOLD-MRI cannot distinguish. Oxygen delivery is primarily influenced by the delivery of blood to the kidneys, which should thus be separately assessed by measurement of RBF or RP. Oxygen consumption requires measurement of oxidative metabolism in the kidneys. Since measurement of RBF by para-aminohippurate clearance is time-consuming, invasive, and lacks single kidney values, several MRI modalities have been developed that allow measurement of RBF or RP with highly detailed anatomical information. DCE-MRI is a well-established method to assess tissue perfusion and has been validated to assess RBF, GFR, and cortical- and medullary blood volumes. Disadvantage is that its use is restricted in patients with severe renal impairment. MRI techniques without contrast agent are therefore better suited for RBF measurement, such as PC-MRI which is used in clinical practice and in CKD populations. H_2_^15^O PET imaging is considered the gold standard for cerebral and myocardial perfusion imaging and is validated to measure RBF. Despite its limited use in nephrology so far, its proven capacity to detect acute changes in RBF offers an attractive alternative to MRI-based modalities when investigating SGLT2i-induced RBF changes. To measure renal tissue perfusion, ASL-MRI is highly suited for taking repeated measures and therefore to assess cortical changes induced by drugs, although the reproducibility in the medulla is moderate to poor. Oxygen consumption can be assessed with [^11^C]-acetate PET imaging. As SGLT2i seem to improve renal oxygenation without increasing RBF, this modality has great potential to provide unique information regarding the effects of SGLT2i on hypoxia.

PET imaging with various radiolabeled substrates are currently used to quantitate the glucose blocking potential of SGLT2i on SGLT1 and SGLT2. However, as the substrates lack the power to discriminate between the specific transporter subtypes, it makes this approach less suitable to quantitate SGLT2 function *in vivo*. PET imaging with a radiolabeled SGLT2i circumvents this issue due to its specific affinity for SGLT2. The radiolabeled SGLT2i should preferable be an isotopologue of its marketed gliflozin, in order to fully reflect its pharmacological properties. With this approach it becomes possible to quantify the tissue pharmacology *in vivo*, and consequently response variability in patients. A feasibility study for the use of [^18^F]-canagliflozin in T2DM patients is currently ongoing (Netherlands Trial Register; NL7707).

Beyond the scope of this mini-review, several PET- and MRI tracers are in preclinical development (Harada et al., 2013) that allow exploration of the suggested SGLT2i-effects on mitochondrial metabolism (Mulder et al., 2019; Ferrannini et al., 2016). In T2DM patients, the anaerobic glycolysis, glucose oxidation and fatty acid oxidation in the heart was assessed by hyperpolarized [^13^C]-pyruvate-MRI (Rider et al., 2020).

A few studies have already shown exciting findings by using different imaging modalities. By combining ASL-MRI, PC-MRI, and BOLD-MRI, an acute SGLT2i-induced improvement in cortical oxygenation in T1DM patients was not accompanied by changes in RP or RBF (Laursen et al., 2021). Also, in T2DM patients, ASL-MRI and BOLD-MRI showed improved cortical and medullary oxygenation after 24-weeks SGLT2 inhibition, without a change in RP (Zhou et al., 2021). Firstly, the suggestion that this must result from reduced oxygen consumption could not be drawn with sole use of BOLD-MRI. Secondly, it is highly interesting to see what actual measurement of renal oxygen consumption by [^11^C]-acetate PET on top of BOLD-MRI and ASL-MRI would add to this, as is currently assessed in an ongoing clinical trial with ertugluflozin in T2DM patients (NCT04027530).

The papers and current trials show the recent developments in renal imaging modalities. We have the tools to assess various effects of SGLT2i on renal oxygenation and hypoxia, fibrosis and their transporter interactions *in vivo* in patients. Combined measurement of RBF/RP by PC-MRI and/or ASL-MRI, oxygen consumption by [^11^C]-acetate PET and oxygenation by BOLD-MRI would provide a wealth of information on the hypoxia effects of SGLT2i. DWI-MRI can assess the benefit of SGLT2 inhibition on renal fibrosis and PET imaging with radiolabeled drugs holds the promise to understand the response-variability between patients. Also, early identification of hypoxia can result in early intervention and thus timely prevention of kidney disease progression. We conclude that in particular the complementary value of multiple imaging modalities in a single clinical trial aids in an integrated understanding of the pharmacology of SGLT2i.
